# Correction: Sun et al. Generation of the Chondroprotective Proteomes by Activating PI3K and TNFα Signaling. *Cancers* 2022, *14*, 3039

**DOI:** 10.3390/cancers14184393

**Published:** 2022-09-09

**Authors:** Xun Sun, Ke-Xin Li, Marxa L. Figueiredo, Chien-Chi Lin, Bai-Yan Li, Hiroki Yokota

**Affiliations:** 1 Department of Pharmacology, School of Pharmacy, Harbin Medical University, Harbin 150081, China; 2 Department of Biomedical Engineering, Indiana University Purdue University Indianapolis, Indianapolis, IN 46202, USA; 3 Department of Basic Medical Sciences and Interdisciplinary Biomedical Sciences Program, Purdue University, West Lafayette, IN 47907, USA; 4 Indiana Center for Musculoskeletal Health, Indiana University School of Medicine, Indianapolis, IN 46202, USA; 5 Simon Cancer Center, Indiana University School of Medicine, Indianapolis, IN 46202, USA

In the original article [1], there was a mistake in Panel E of Figure 2 as published. The original images for the TNFα case were mistakenly used. The corrected Figure 2 appears below.

The authors apologize for any inconvenience caused and state that the results and scientific conclusions are unaffected. This correction was approved by the Academic Editor. The original publication has also been updated.

## Figures and Tables

**Figure 2:**
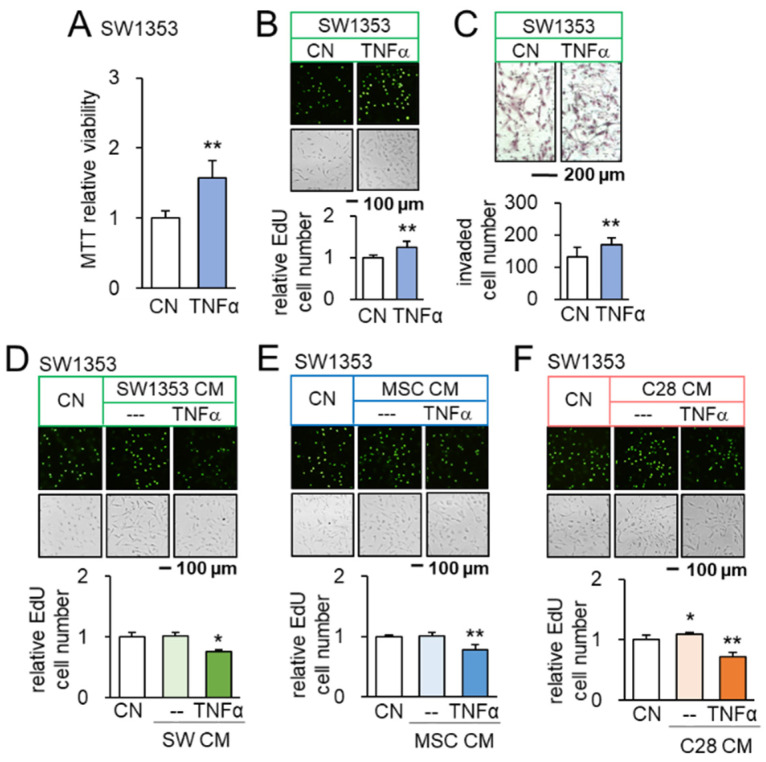
Opposing effects of TNFα and TNFα-treated CM on SW1353 CS cells. CN = control, CM = conditioned medium, and C28 = C28/I2 chondrocytes. The single and double asterisks indicate *p* < 0.05 and 0.01, respectively. (**A**–**C**) Increase in MTT-based viability, EdU-based proliferation, and transwell invasion of SW1353 cells in response to 10 ng/mL of TNFα (*n* = 6). (**D**) Upregulation of Runx2 and Snail in SW1353 cells by 10 ng/mL of TNFα (*n* = 6). (**E**,**F**) Reduction in EdU-based proliferation of SW1353 cells by TNFα-treated CS cells, MSC, and C28 chondrocyte-derived CMs, respectively (*n* = 6). The concentration of TNFα was 10 ng/mL.
