# Supplementary material for: Generation of the Chondroprotective Proteomes by Activating PI3K and TNFα Signaling
Source: Cancers (Basel). 2022 Jun 21;14(13):3039. doi: 10.3390/cancers14133039 (PMC9264838; doi:10.3390/cancers14133039)
Supplement: Supplementary file 1 [file cancers-14-03039-s001.zip › cancers-1733718-supplementary.pdf]

Supplementary Materials

Generation of the Chondroprotective Proteomes by Activating PI3K and TNFα Signaling

Xun Sun, Ke-Xin Li, Marxa L. Figueiredo, Chien-Chi Lin, Bai-Yan Li and Hiroki Yokota

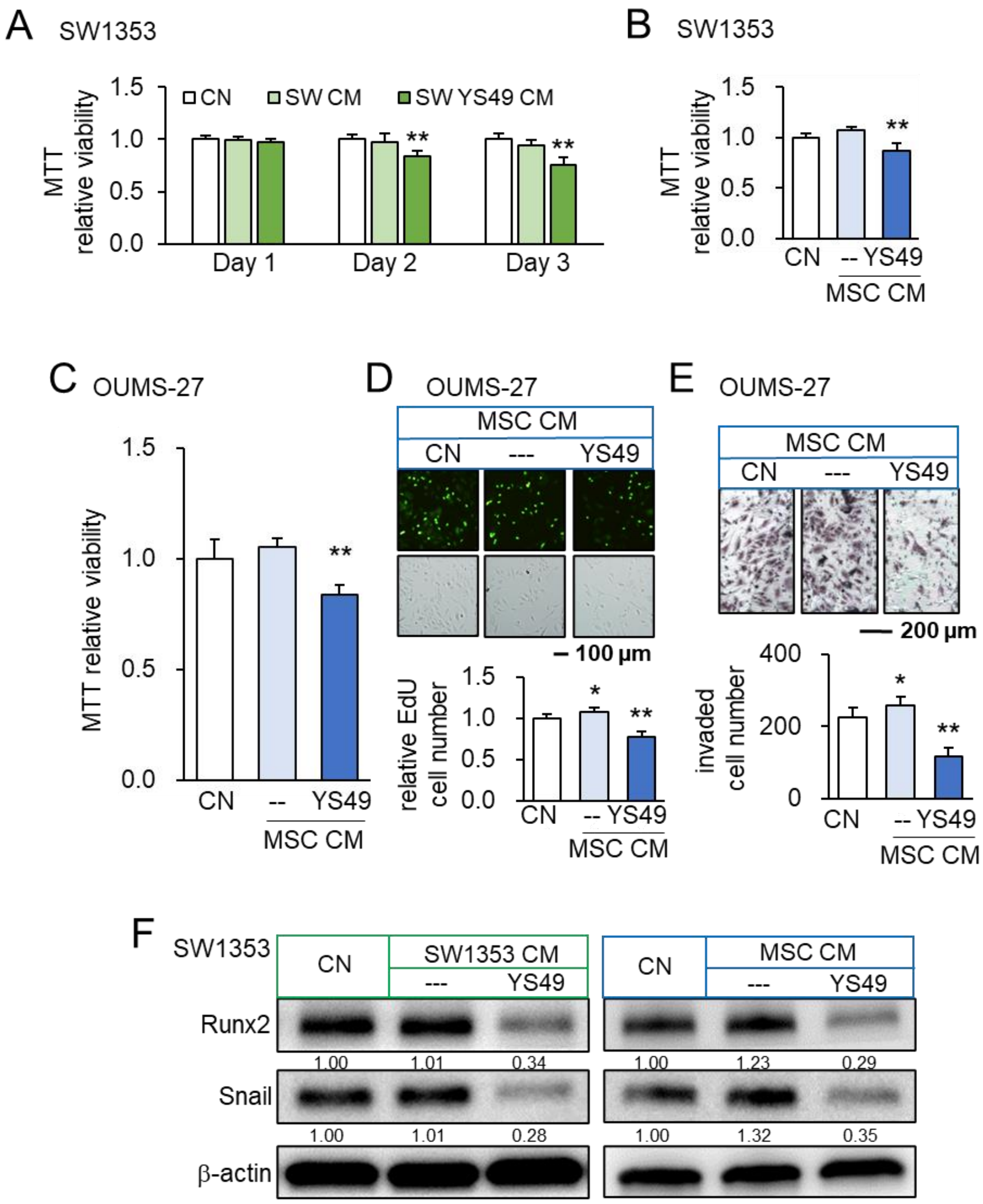

Figure S1. Anti-tumorigenic effects of YS49-treated SW1353 and MSCs-derived CM. CN = control, and CM = conditioned medium. The single and double asterisks indicate $p < 0.05$ and 0.01, respectively. (**A–B**) Reduction in MTT-based viability of SW1353 chondrosarcoma cells by YS49-activated SW1353 and MSC-derived CM. (**C–E**) Reduction in MTT-based viability, EdU-based proliferation, and transwell invasion of OUMS-27 chondrosarcoma cells by YS49-treated MSC-derived CM. (**F**) Downregulation of Runx2 and Snail in SW1353 cells by YS49-treated chondrosarcoma/MSC-derived CM. Full Western blot images are available in Figure S8.

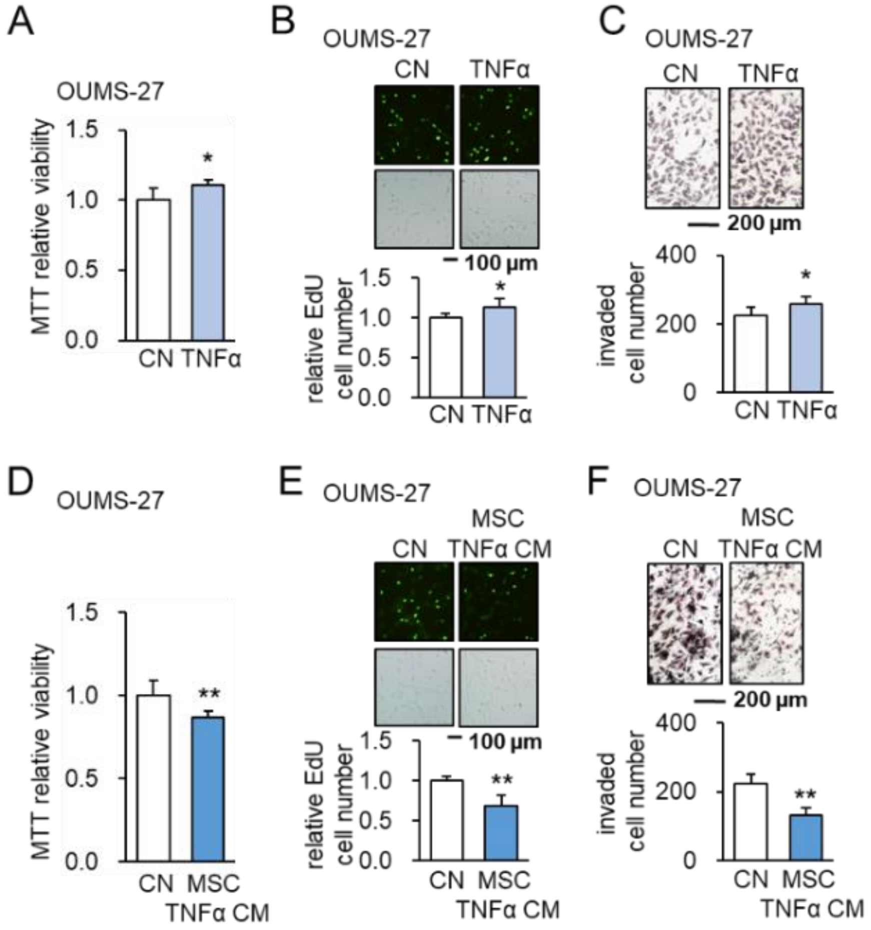

Figure S2. Anti-tumorigenic effects of TNFα-treated MSCs-derived CM. CN = control, CM = conditioned medium, and OUMS-27 = OUMS-27 chondrosarcoma cells. The single and double asterisks indicate $p < 0.05$ and 0.01, respectively. (**A–C**) Increase in MTT-based viability, EdU-based proliferation, and transwell invasion of OUMS-27 cells by 10 μg/ml of TNFα. (**D–F**) Reduction in MTT-based viability, EdU-based proliferation, and transwell invasion of OUMS-27 cells by TNFα-treated MSCs-derived CM.

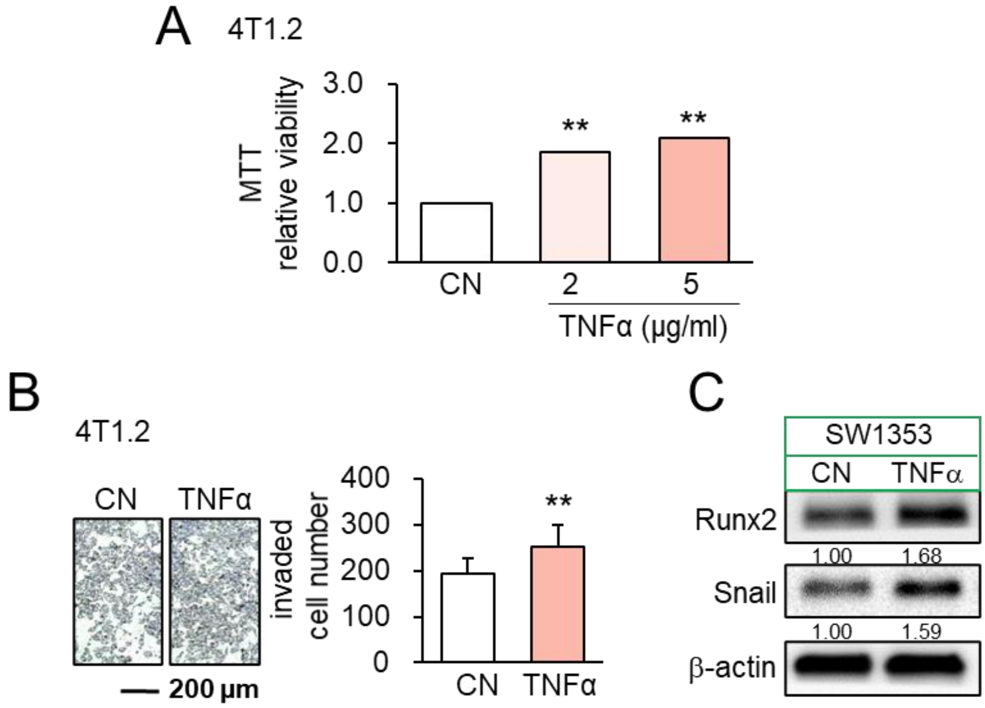

Figure S3. The tumor-promoting effect of TNFα cytokine on 4T1.2 mammary tumor cells. (**A**,**B**) Promotion of MTT-based viability and transwell invasion of 4T1.2 cells in response to 2 and 5 μg/ml of TNFα. (**C**) Upregulation of Runx2 and Snail in SW1353 cells in response to 5 μg/ml of TNFα. Full Western blot images are available in Figure S8.

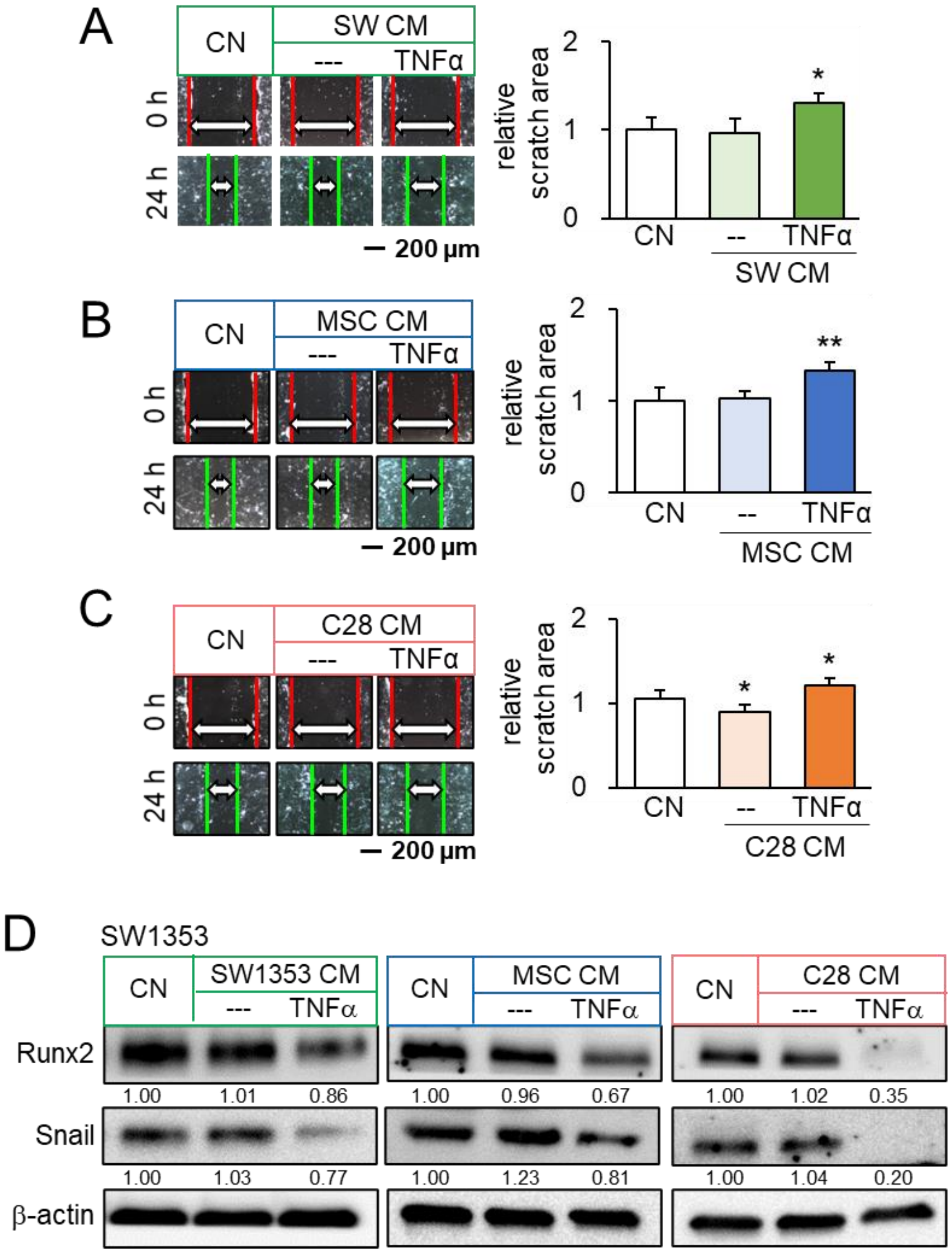

Figure S4. Anti-motility effects of TNFα-treated CM. CN = control, CM = conditioned medium, SW = SW1353 chondrosarcoma cells, and C28 = C28/I2 chondrocytes. The single and double asterisks indicate $p < 0.05$ and 0.01. (**A**–**C**) Reduction in scratch-based motility of SW1353 cells by TNFα-treated chondrosarcoma cell, MSC, and C28 chondrocyte-derived CMs, respectively. (**D**) Downregulation of Runx2 and Snail in SW1353 cells by TNFα-treated chondrosarcoma cell, MSC, and C28 chondrocytes-derived CM, respectively. Full Western blot images are available in Figure S8.

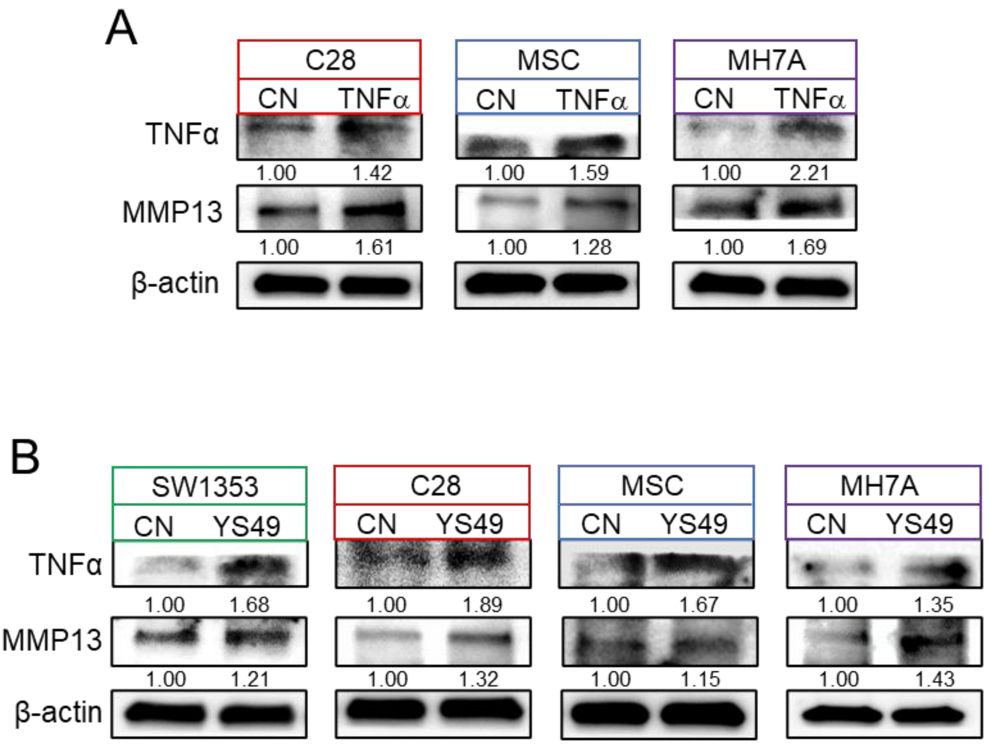

Figure S5. Upregulation of TNFα and MMP13 in C28/I2 chondrocytes in response to TNFα and YS49. (**A**) Upregulation of TNFα and MMP13 of chondrocytes, MSC cells, and MH7a cells in response to 10 μg/ml TNFα. (**B**) Upregulation of TNFα and MMP13 in SW1353 cells, C28 cells, MSC cells, and MH7a cells in response to 50 μM of YS49. Full Western blot images are available in Figure S8.

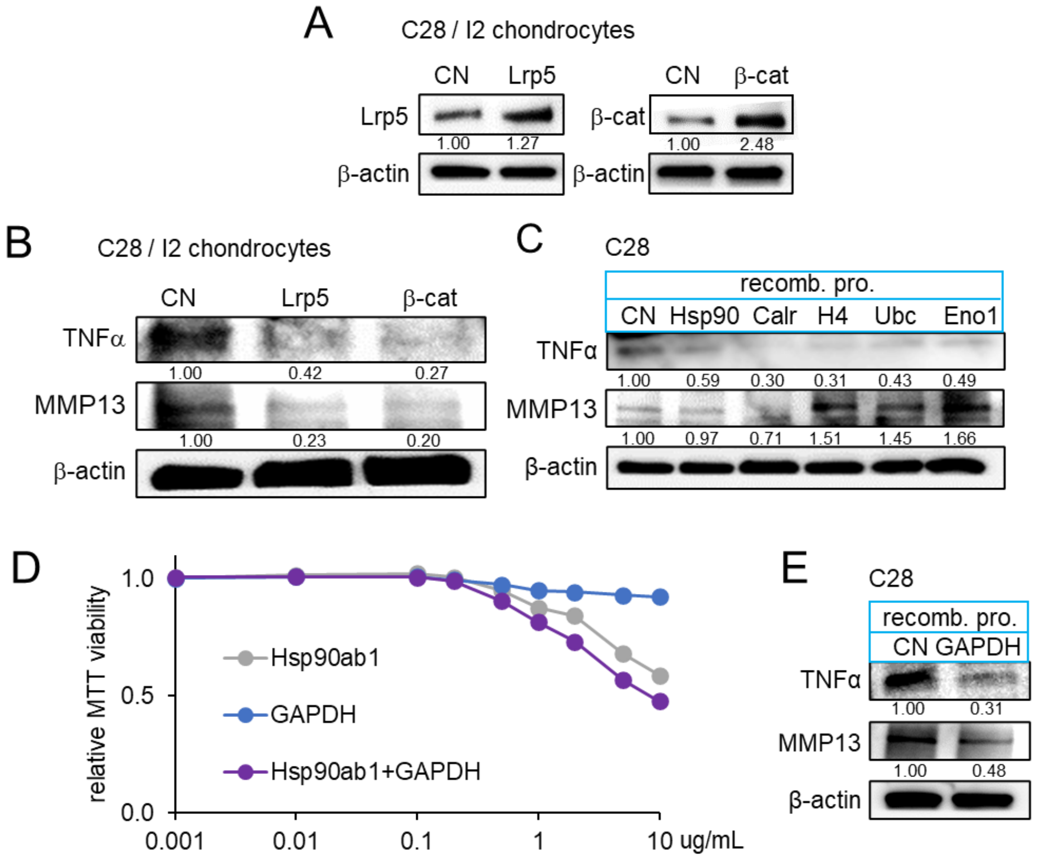

Figure S6. Downregulation of TNFα and MMP13 in C28/I2 chondrocytes by Lrp5-overexpressing C28 cell-derived CM, Hsp90ab1 and GAPDH. CN = control, Lrp5 = Lrp5 overexpression, and β-cat = β-catenin overexpression. (**A**) Overexpression of Lrp5 and β-catenin in C28/I2 chondrocytes. (**B**) Downregulation of TNFα and MMP13 in response to Lrp5/ β-catenin-overexpressing C28 cell-derived CM. (**C**) Expression of TNFα, MMP13, and FoxO1 in C28/I2 chondrocytes in response to recombinant 1 μg/mL of Hsp90ab1 (HSP), 0.8 μg/mL of Calreticulin (Calr), 1 μg/mL of histone H4 (H4), 0.8 μg/mL of polyubiquitin C (Ubc), and 0.8 μg/mL of enolase 1 (Eno1). (**D**) MTT-based dose responses of GAPDH and Hsp90ab1 in SW1353 chondrosarcoma cells. (**E**) Downregulation of TNFα and MMP13 in C28/I2 chondrocytes in response to recombinant 2 μg/mL of GAPDH. Full Western blot images are available in Figure S8.

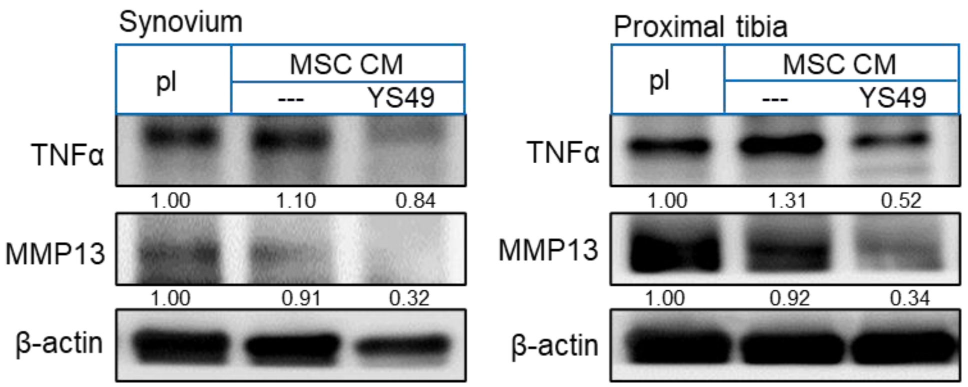

Figure S7. Downregulation of TNFα, IL1β, and MMP13 in the synovium and the proximal bone of tumor-inoculated NSG mice in response to YS49 CM. Full Western blot images are available in Figure S8.

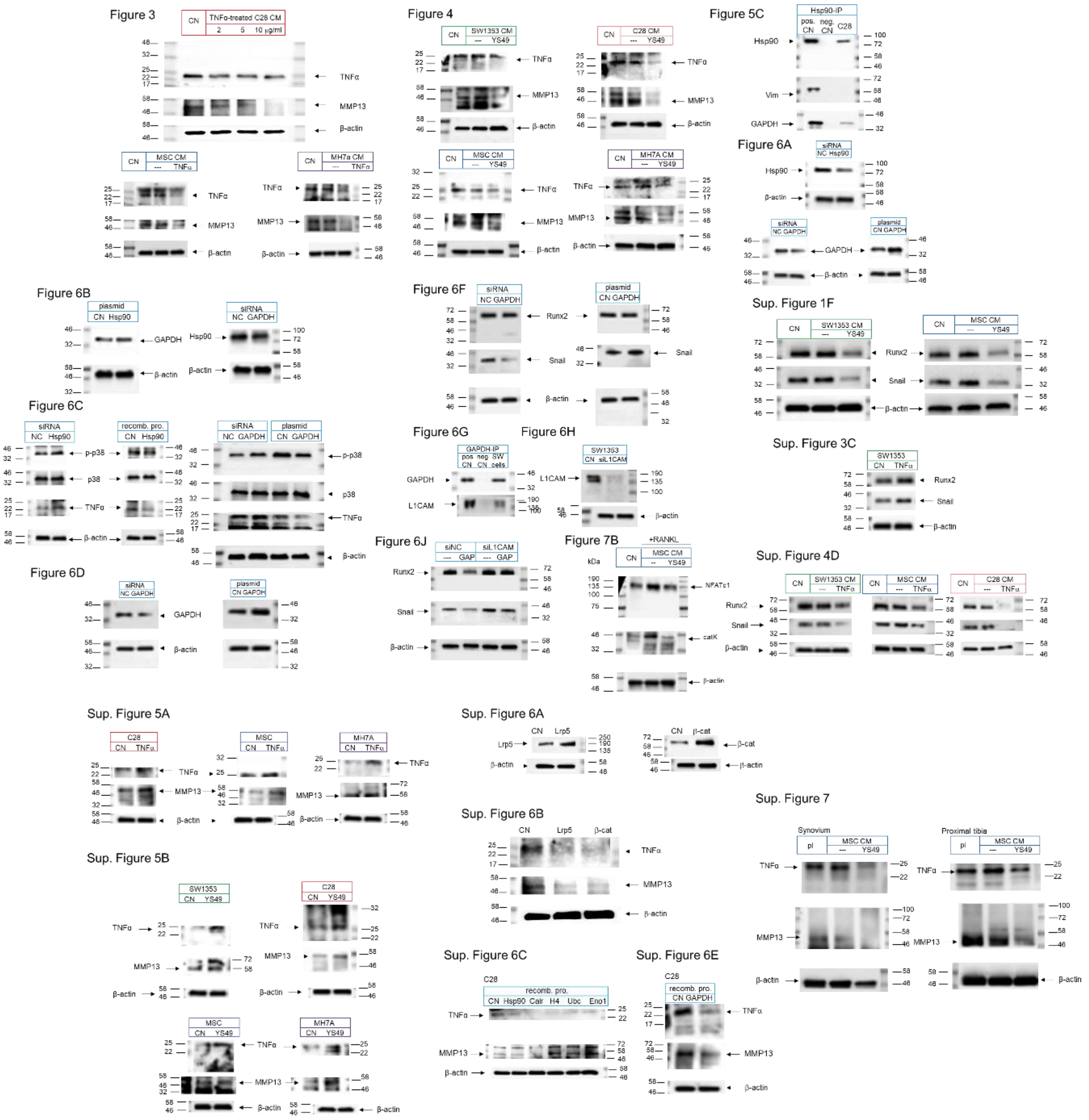

Figure S8. Full Western blot images.

Table S1. List of 39 proteins that were immunoprecipitated with Hsp90ab1 in mass spectrometry-based proteomics analysis. The score indicates the MS/MS counts, which represent the relative protein abundance.

#	Gene Name	Score	#	Gene Name	Score	#	Gene Name	Score
1	*Vim*	113	14	*Serpinh1*	35	27	*Ncl*	27
2	*Hspa5*	80	15	*Myh9*	32	28	*Ahnak*	26
3	*Stip1*	80	16	*Calr*	32	29	*Ubc*	26
4	*Hspa8*	78	17	*Tubb4b*	30	30	*Calu*	26
5	*Hspd1*	70	18	*Hnrnpk*	29	31	*Eef2*	25
6	*P4hb*	59	19	*Actb; Actg1*	29	32	*Hspe1*	23
7	*Pdia3*	57	20	*Canx*	29	33	*Rpl4*	23
8	*Hspa9*	50	21	*C1qbp*	29	34	*Fkbp4*	23
9	*Atp5b*	47	22	*Npm1*	29	35	*Tuba1b*	22
10	*Gapdh*	44	23	*Hap90aa1*	29	36	*Hnrnpa1*	21
11	*Atp5a1*	41	24	*Hnrnpa3*	28	37	*Pabpc1*	21
12	*Hnrnpa2b1*	39	25	*Mybbp1a*	28	38	*Anxa2*	20
13	*Hsp90b1*	37	26	*Eef1a1*	27	39	*Khsrp*	20
